# Application of Carbon Nanoparticles in Oncology and Regenerative Medicine

**DOI:** 10.3390/ijms22158341

**Published:** 2021-08-03

**Authors:** Katarzyna Lisik, Anita Krokosz

**Affiliations:** 1 Faculty of Biology and Environmental Protection, University of Lodz, 90-236 Lodz, Poland; kasialisik@interia.pl; 2 Department of Biophysics of Environmental Pollution, Faculty of Biology and Environmental Protection, University of Lodz, 90-236 Lodz, Poland

**Keywords:** fullerenes, nanotubes, nanodiamonds, cancer, theranostics, bone reconstruction

## Abstract

Currently, carbon nanoparticles play a large role as carriers of various types of drugs, and also have applications in other fields of medicine, e.g., in tissue engineering, where they are used to reconstruct bone tissue. They also contribute to the early detection of cancer cells, and can act as markers in imaging diagnostics. Their antibacterial and anti-inflammatory properties are also known. This feature is particularly important in dental implantology, where various types of bacterial infections and implant rejection often occur. The search for newer and more effective treatments may lead to future use of nanoparticles on a large scale. In this work, the current state of knowledge on the possible use of nanotubes, nanodiamonds, and fullerenes in therapy is reviewed. Both advantages and disadvantages of the use of carbon nanoparticles in therapy and diagnostics have been indicated.

## 1. Introduction

Today, nanotechnology is one of the most dynamically developing sciences. Increasingly, nanotechnological achievements are used in the biological and medical fields. This application is possible due to the production of nanoparticles, where the size does not exceed 100 nm [1,2,3]. Considering their origin, nanoparticles can be divided into: Natural and designed, and considering their structure, into spherical, fibrous and layered [4]. Natural nanoparticles are formed in nature as a result of, for example, the erosion of geological materials, as well as decomposition of biological materials, mainly plant residues. They can also be produced as a result of combustion of fuel products [5,6,7]. Designed nanoparticles are manufactured by the nanotechnology industry. They are characterized by variable physical and chemical properties, a specific size and various modifications of their surface. Due to these modifications, they demonstrate very good adsorption and absorption properties, and are also able to aggregate. All these features of nanoparticles make them useful in many fields, e.g., nanomedicine, nanopharmacology and nanooncology. All these properties are met by carbon nanoparticles. In this work, various reports on carbon nanoparticles: Fullerenes, nanotubes, and nanodiamonds, and their application in some diseases are reviewed to assess the advantages and disadvantages of these nanoparticles in the light of the latest biomedical research.

## 2. Characteristics of Fullerenes, Nanotubes and Nanodiamonds

### 2.1. Fullerenes

**Figure 1:**
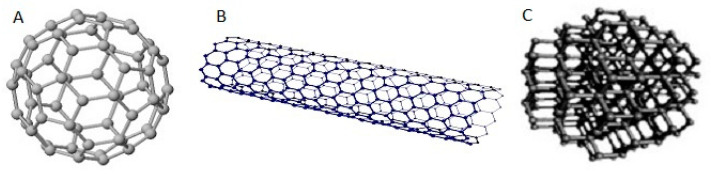
Structures of (**A**): C_60_ fullerene (http://chemia.waw.pl/fulereny.htm accessed on 13 June 2021); (**B**): carbon nanotube (http://www.fizyka.iss.com.pl/nanorurki/08rysunki05.html); (**C**): nanodiamond [10].

Fullerenes are the third allotropic form of carbon, in which carbon atoms form the shape of a regular and hollow sphere or ellipse. The empty space inside makes it able to accommodate other atoms, including radioactive atoms important in diagnostics. Fullerenes can vary in size. The largest are composed of as many as 1500 carbon atoms [1], and the smallest of 20. However, existence of C20 fullerene is only theoretical, because not all fullerenes are chemically stable enough. Among the abundant group of fullerenes, the most famous are those containing 60 and 70 carbon atoms, and the most stable structure is that of C60 fullerene (Figure 1A). The C60 particle is made up of 20 hexagonal faces and 12 pentagonal faces. There are two types of bonds: Single C5-C5 in pentagons and double C5-C6 in hexagons. Delocalization of 30 double bonds and sharing of all π electrons gives the molecule a high stability. Due to their hydrophobic properties, fullerenes show poor solubility in polar liquids [8,9].

Unfortunately, the high hydrophobicity of carbon nanoparticles and the ability to form aggregates in aqueous solvents were obstacles in their use in medicine [11]. The problems were solved by modifying the surface of carbon molecules by introducing hydrophilic functional groups. The more hydrophilic groups, the greater the solubility of the molecule in aqueous solutions [11,12,13]. These modifications, apart from ensuring better solubility, also reduce the toxicity of carbon nanoparticles [14,15,16]. The second method of modification masks the carbon coating with dextrins, forming complexes with fullerenes and thus increasing the solubility of the resulting derivatives in polar solvents [17].

### 2.2. Nanotubes

Another group of carbon nanoparticles with promising properties for use in nanomedicine are carbon nanotubes (NTs). They were discovered in 1992. They have the shape of a cylinder made of layers of folded graphene (Figure 1B). Inside these structures there are empty spaces, like in the C60. This feature allows transporting active substances. The high ratio of length to diameter of these particles allows attachment of many active substances with different functions, and controlling their transport [9,18,19]. Considering the number of layers building the nanotube wall, carbon nanotubes are divided into: single-walled (SWCNTs) and multi-walled (MWCNTs). These particles, like fullerenes, are hydrophobic, which results in their low solubility in aqueous solutions.

Therefore, they must also undergo certain modifications. Such functionalizations are performed using two methods: Exohedral and endohedral. The first method uses structural defects occurring on the side walls of nanotubes and at their ends, and the second method consists in filling the empty space with various polar substances [20,21].

Exohedral functionalization can be carried out by attaching substances with covalent or non-covalent bonds. In non-covalent functionalization, substances interact with the nanotube through π-π bonds or Van Der Waals forces [9,22]. The task of covalent functionalization on the defects and side walls is to permanently attach individual compounds to the nanotube surface [23]. Functionalization on defects occurs mainly on -COOH groups. Functionalization of sidewalls takes place directly on carbon atoms of the nanotube and consists in the addition to double bonds, causing a change of carbon atoms hybridization from sp2 to sp3, which causes a significant change in the electron properties of the nanotube [20]. Functionalized NTs can be used as diagnostic tools for early detection of cancer [24]. Recent studies show that functionalized NTs can cross the blood-brain barrier [25,26].

### 2.3. Nanodiamonds

Nanodiamonds (NDs) are tiny diamond crystals only a few nanometers in diameter, composed of carbon atoms arranged regularly (Figure 1C). They show exceptional hardness and excellent wear resistance [27,28,29]. Compared to fullerenes and nanotubes, NDs have a rough surface which facilitates their adhesion to other molecules. Due to their properties and the ease of surface modification with various compounds, nanodiamonds are a promising material for use in medicine as drug transporters [30]. In vitro and in vivo studies have shown that NDs are well tolerated by biological systems [31]. For example, the research conducted on mice with liver cancer given the ND-doxorubicin (DOX) conjugate showed that DOX was released into cells more slowly compared to the time noted after dosing with DOX alone. A 4-fold longer survival time of mice with liver cancer treated with the ND-DOX conjugate was also observed. Moreover, doxorubicin in the form of ND-DOX showed no toxic effect on kidneys, the liver and the spleen, in contrast to the free drug [32].

Nanodiamonds have now received considerable attention due to their excellent properties and a variety of applications, especially in the field of biomedicine [33]. Nevertheless, the tendency to form clumps restricts further extensive use of nanodiamonds. Guo et al. [34] examined the size of these molecules after attaching natural amino acids and ascorbic acid to their surface. They found that nanodiamonds functionalized by biomolecules had smaller particle size, demonstrated a lower aggregation and formed stable dispersions in organic and aqueous solutions [34]. This can increase the use of nanodiamonds especially in the biological field.

## 3. Carbon Nanoparticles in Cancer and Bone Reconstruction

### 3.1. Targeted Therapy and Theranostics in Cancer

Medicine is constantly looking for more and more perfect methods of treatment. Due to their properties, very small size and ease of surface functionalization, carbon particles are promising, inter alia, in the treatment of neoplastic diseases [35,36,37,38]. The treatment and diagnostics of cancer are currently one of the most important problems in medicine [39].

Due to the use of nano-scale molecules, it is possible to improve the treatment of various types of cancer through targeted therapy or early diagnostics. In targeted therapy, there is an interaction with signaling proteins of cancer cells. Drugs block cell receptors responsible for transmission of a signal activating cancer cells for proliferation and thus lead to its programmed death (apoptosis). A modern solution used in targeted therapy is the use of monoclonal antibodies directed against specific antigens on the surface of a cancer cell, thereby achieving the intended therapeutic effect [40]. Selective drug delivery directly to tumor cells significantly reduces the risk of exposing normal cells and tissues [41,42]. In in vivo colon cancer studies, conjugates of nanodiamonds with chemotherapeutic agents: ND-cisplatin and ND-cetuximab combined with monoclonal antibodies against the epidermal growth factor receptor (EGFR) showed selective binding of the conjugate to antibody-bearing cancer cells. Increased therapeutic efficacy of this conjugate has also been demonstrated in human liver cancer cell lines [43]. Further research in this direction offers very promising prospects for the use of monoclonal antibodies in cancer therapy.

**Scheme 1:**
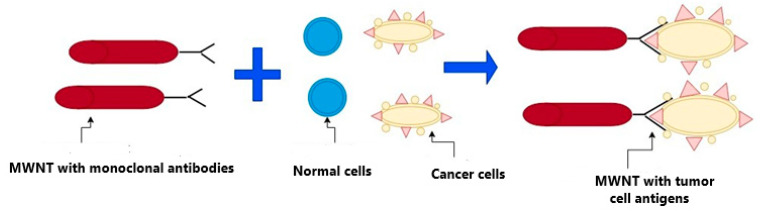
Carbon nanotubes coated with monoclonal antibodies binding to antigens of cancer cells.

Theranostics is the most modern method of treatment combining diagnostics and therapy. It allows the precise selection of the treatment targeted at an individual patient. Fullerenes, nanotubes and nanodiamonds are used as carriers of various drugs, enabling their accurate and longer lasting application in cancer-altered tissue. Welsher et al. [44] investigated the possibility of using nanotubes as fluorescent markers used as SWCNTs sensors for the recognition of cancer cells. For this purpose, single-walled nanotubes combined with appropriate monoclonal antibodies (Rituxan and Herceptin) were used and mixed with normal and cancer cells. These antibodies recognize CD20 cell surface receptors and the HER2/neu receptor on breast cancer cells. It was observed that the nanotubes with attached antibodies bound to cancer cell receptors. The mixture was additionally irradiated with 785 nm wavelength radiation. This resulted in photoluminescence of the nanotubes in the infrared range. This method allowed to distinguish between normal and cancer cells [44,45,46,47]. The specific binding of antibodies-labelled nanotubes to antigens of cancer cells is shown in Scheme 1.

Often, the effect of anti-cancer drugs is limited by its poor water solubility. This is the case, for example, of the chemotherapeutic agent Paclitaxel (PTX) used to treat breast cancer. In order to change the solubility of PTX, Shao et al. [38] investigated the modification of SWCNTs by coupling SWCNTs with human serum albumin (HSA). In this way, the SWCNT-HSA complex was created, which was used as the carrier for PTX. It was observed that this complex was very efficiently delivered to MCF-7 breast cancer cells (cell uptake rate of 80%). The administration of PTX on the SWCNT-HSA vehicle inhibited the growth of MCF-7 tumor cells more efficiently than PTX coupled with HSAP only. The tumor cell survival rate decreased from 70% to 63% after 48 h of cell incubation with PTX-HSA and SWCNT-HSA-PTX, respectively. The difference in survival in favor of greater efficacy of SWCNT-HSA-PTX was maintained for 72 h of incubation. The SWCNT-HSA-PTX conjugate showed a stronger anti-tumor effect than HSA-PTX [38]. Therefore, the combination of carbon nanotubes with plasma albumin seems greatly promising in the delivery of cancer drugs to cancer cells.

Ji et al. [41] tested the conjugate of carbon nanotube coated with chitosan with folic acid and doxorubicin (DOX/FA/CHI/SWCNT) at the concentration of 50 and 100 µg/mL administered intravenously to mice with liver cancer. The arrest of the development of cancer cells was found for both concentrations, compared to free DOX (100 µg/mL) in nude BALB/C mice. The pharmaceutical efficiency was further examined by measuring both the viability of SMMC-7721 cells in vitro and the tumor volume of liver cancer in nude mice in vivo after treatment with the DOX/FA/CHI/SWCNTs. In addition, routine blood tests, serum biochemistry parameters, and histological examinations were also used to study the in vivo toxicity of the new drug delivery system (DDS) [41]. This “targeted conjugate” showed higher levels of cytotoxicity and apoptotic activity than free doxorubicin.

Diamond nanoparticles (NDs) are highly biocompatible [30,48] and relatively safe, making them a very promising material for medical applications. These particles are hoped to deliver drugs to the tumor area. ND, like multi-wall carbon nanotubes (MWCNTs), can form complexes with poorly water-soluble chemotherapeutic agents such as anthracyclines. These drugs bind non-covalently with NDs having hydroxyl or carboxyl groups on their surfaces [49]. They cause the drug to remain in a specific place for a longer time, which leads to an increase in the sensitivity of cancer cells to the drug [33,50]. NDs are also characterized by low toxicity towards normal cells. These complexes allow the drug to be slowly released for at least one month, with an adequate amount of drug in reserve [48]. The slow release of the drug means fewer doses of chemotherapy for the patient, and therefore fewer side effects of the chemotherapy.

One method of silencing expression of a defective gene is to deliver interfering RNA (siRNA) to tumor cells. Nanodiamonds of 35 nm are coated with polyethyleneimine to efficiently deliver siRNA to cells, resulting in inhibition of EWS/FLI1 oncogene expression in Ewing’s sarcoma. The siRNA/ND complex with the anti-cancer drug Vincristine increases the toxic effect of this drug on cancer cells [51]. However, there are some obstacles that limit the usefulness of siRNAs, namely instability of the molecule, poor cellular uptake, and too large a size. All of this hinders penetration of siRNA into cells by diffusion [52,53]. In preclinical studies, human HDL lipoprotein fraction was used to transport siRNA. Shahzad et al. [53] showed that nanoparticles with the HDL fraction could efficiently deliver STAT3 siRNA and FAK siRNA to the HCT116 mouse cell line. In other siRNA studies, Bi et al. [54] developed a new system for delivering siRNA by the survivin-siRNA conjugate and the donor of this conjugate—NDCONH (CH2) 2NH-VDGR—containing ND (200 mg). Human MCF-7 breast cancer cells were treated with survivin-siRNA and conjugates also containing nanoparticles (NDCONH (CH2) 2NH-VDGR/survivin-siRNA) and grown for 48 h. Stained MCF-7 cells were analyzed with a scanning microscope. Green fluorescence (FAM-survivin-siRNA) was observed around the nucleus of MCF-7 cells treated with NDCONH (CH2) 2NH-VDGR/FAM-survivin-siRNA, compared to the group treated with survivin-siRNA only. It was found that the survivin-siRNA complex could be transferred into the cytoplasm of MCF-7 cells by NDCONH (CH2) 2NH-VDGR [54]. The technique of introducing siRNA by nanoparticles into selected tissues seems to be a promising method of treating many previously incurable diseases.

### 3.2. Reconstruction of Bone and Cartilage

**Scheme 2:**
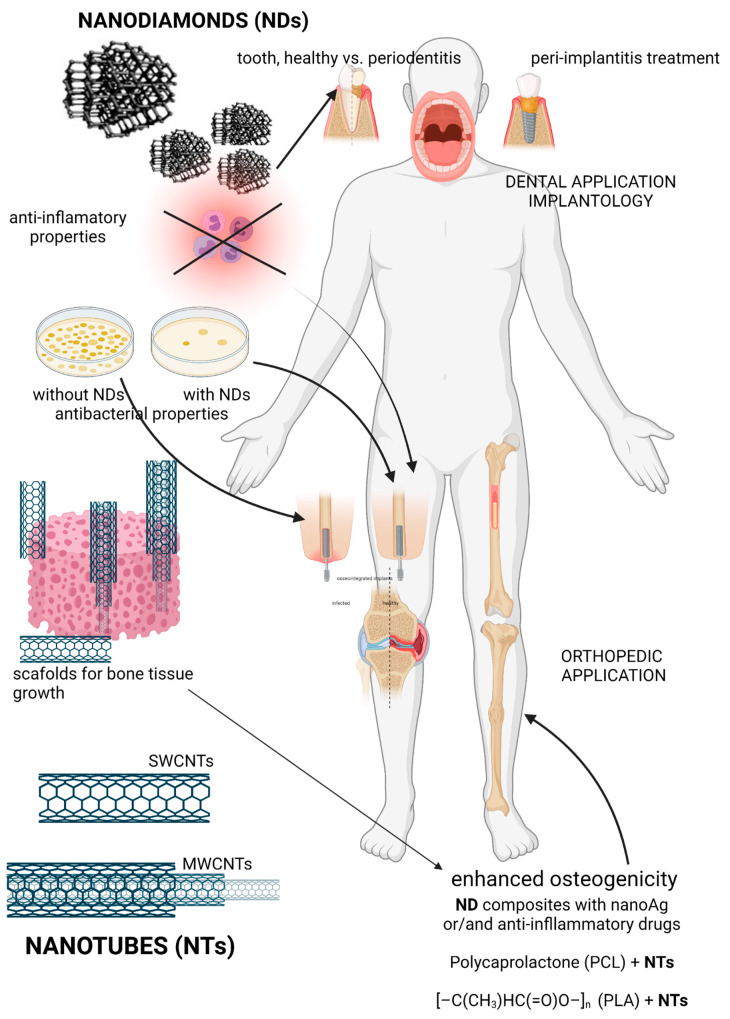
Carbon nanotubes and nanodiamonds in bone reconstruction and dental implantology. Created with BioRender.com accessed on 13 June 2021.

Carbon nanoparticles can also be potentially used in the treatment of other diseases, including in tissue engineering, e.g., for the reconstruction of bone defects. The use of an appropriate type of nanoparticle in this field of medicine can significantly improve the biological and mechanical properties of composite scaffolds for bone systems, and can also have various functions depending on the application [55,56,57]. Due to its mechanical properties, tensile strength and fibrous structure, carbon nanotubes (NTs) have been used in tissue engineering to improve mechanical properties of polymers [58,59]. Magiera et al. [60] investigated the combination of NTc with polylactic acid (PLA). This complex shows a much better biocompatibility than PLA/gelatin nanofibers. PLA/NTs complexes showed a better osteo-induction compared to pure PLA nanofibers. PLA nanofibers contained in NTs improved mechanical properties of hybrid samples, compared to PLA/GEL, and such a 3D system seems to be interesting for potential applications as a composite scaffolding in TE [60]. Multi-walled carbon nanotubes (MWCNTs) have the ability to differentiate stem cells into bone cells and increase bone formation. A MWCNTs-containing scaffold showed slower degradation [61]. In the study on rat bone marrow stromal cells (BMSC), Pan et al. [56] showed that the addition of NTs to polycaprolactone (PCL) improved mechanical properties of PCL. An increase in proliferation and differentiation of bone marrow stem cells was also demonstrated [56]. Scheme 2 shows the use of carbon nanotubes and nanodiamonds in bone reconstruction and dental implantology. Although, these results are promising, one should bear in mind the results of studies indicating the toxicity of nanoparticles discussed in Section 6.

## 4. Antibacterial and Anti-Inflammatory Properties of Nanoparticles in Implantology

A very important aspect in tissue engineering is the reconstruction of cartilage. The main problem in this process is finding right materials by which cells could be cultured. These media must not be toxic to cultured cells and must have antibacterial properties. This is where nanodiamonds (ND) play their role perfectly. Ibrahim et al. [55] conducted studies on human SaOS-2 osteoblast cells and U937 monoblastoid cells exposed to nanodiamonds of various diameters in the range of 2.36-4.42 nm for 24 h and in five different concentrations (0.002-2 mg/l). The researchers showed that nanodiamond particles improved osteogenicity of SaOS-2 cells without apparent cytotoxicity, but concentration-dependent cytotoxic and inflammatory responses were observed in U937 cells, which negatively affected osteogenicity in cultures [55]. In the study conducted by Wang et al. [62], antibacterial nanodiamond composites (QND, QND-Ag) were used to modify polyurethane (APU) used in culture media. Polyurethane containing QND-Ag provided a mechanical support to the substrate and showed excellent antibacterial activity against Staphylococcus aureus. Antibacterial properties of nanoparticles are also used in dental implantology, where traditional implants are often rejected, and bacterial infections and inflammations occur. It was also found that not only antibacterial properties, but also the properties of nanoparticles as drug carriers, are used in dental implantology. The structure of nanotubes allows placement of anti-inflammatory drugs, e.g., naproxen. Researchers have proven that the drug can be gradually released after the procedure, preventing inflammation around the implant [63]. A group of scientists from UCLA has shown that ND nanoparticles were ideal for root canal treatment. Combined with gutta-percha and amoxicillin, they are a very good filling material. Tests have shown that the preparation works well in teeth with atypical anatomical structure, e.g., teeth with narrow, obstructed canals, curved roots, or atypical side branches [63,64].

## 5. Transport of Carbon Nanoparticles into Cells

### 5.1. Transport of Nanoparticles to Cancer Cells

**Scheme 3:**
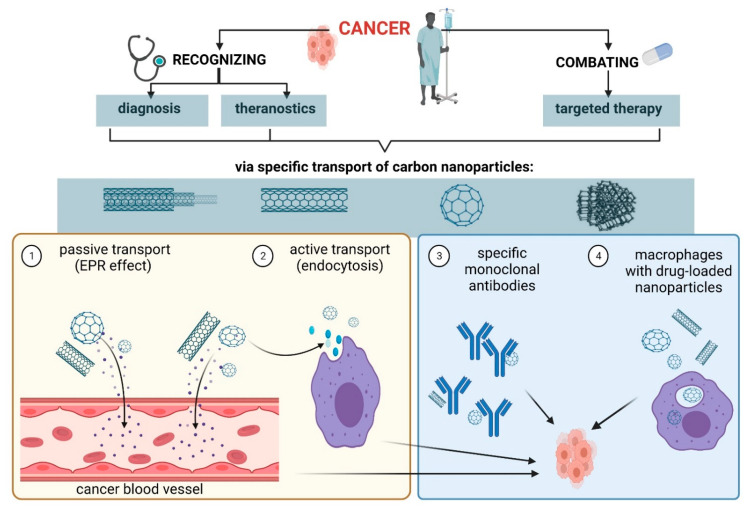
Transport of carbon nanoparticles into cancer cells. Created with BioRender.com accessed on 13 June 2021.

As carbon nanoparticles are a promising material to be used in medicine, mainly as drug carriers, in order to recognize and destroy cancer cells, it is important to understand how specific agents are delivered to tumor areas. Most nanoparticles are able to transport drugs to tumor cells in a passive manner, taking advantage of selectively increased permeability and retention of tumor vessels [42], or by active transport through endocytic pathways. Molecules coated with various ligands bind to cell receptors and penetrate inside a cell by endocytosis, thus, delivering a higher concentration of a drug to the inside of a cancer cell, without causing greater cytotoxicity to normal cells [65]. Possible ways of nanoparticles transport into cancer cells are shown in Scheme 3. All routes of entry are preferred in cancer cells compared to healthy cells due to the differences in the biochemistry of these cells.

The needle-like shape of the NTs allows them to cross the cell membrane as a result of endocytosis and enter cancer cells to deliver the attached drug [36]. Efficacy of ND transport by the endocytic route was investigated using A549 human lung cancer cells and the HFL1 human non-cancerous lung fibroblast cell line, and Beas-2b human bronchial epithelial cells. It was shown that ND penetrated cancer cells to a much greater extent than normal cells, by clathrin-dependent endocytosis [66,67]. Research by Solarska-Ściuk et al. [66] showed that NDs entered the cytoplasm of cells by both clathrin-dependent endocytosis and micropinocytosis, without disturbing the function of endothelial and epithelial cells at concentrations of up to 50 µg/mL after 24 h of incubation. The used cell lines of both human and animal origins allow us to predict the transport of nanoparticles through cell membranes in vivo, and to conduct research on the bioavailability of various drugs. Therefore, they are becoming increasingly used as useful tools for the assessment of active substances.

### 5.2. Passing the Blood-Brain Barrier

The endocytic pathway is essential not only for the transport of drugs but also of nutrients that cross the blood-brain barrier [68]. Research has shown that multi-walled carbon nanotubes (MWCNTs) crossed the blood-brain barrier (BBB). In the work of Gonzalez-Carter et al. [26], functionalized anionic, cationic and non-ionic MWCNTs were examined, in order to investigate the cellular uptake across the blood-brain barrier. the results showed that a large proportion of the cationic and non-ionic, but not anionic, MWCNTs remained in the brain’s endothelial cell membrane. The uptake of MWCNTs by brain endothelial cells is low (<1.5%) and does not correlate with BBB translocation. Anionic MWCNTs have the highest transport levels in the in vitro human BBB model compared to non-ionic or cationic nanotubes [26]. This is confirmed by Moscariello et al. [45] who showed that fluorescent NDs surrounded by a biopolymer coating based on human serum albumin (dcHSA-PEG) were taken up by target brain cells. The use of dcHSA-ND confirms the ability of complexes/conjugates to cross the BBB in a mouse model. Observation of dcHSA-ND is possible at the single cell level and reveals its in vivo uptake into neurons and astrocytes. This study shows NDs penetration into the brain via a BBB transport mechanism [45].

### 5.3. Macrophages in Transport of Nanoparticles

Recently, the use of macrophages as cells for capturing nanoparticles has been particularly interesting [69]. In response to the Abraxan-induced apoptosis of MDA-MB-435 tumor cells in mice, infiltration of macrophages in the tumor region was observed. Those macrophages captured drug-loaded nanoparticles and targeted them to cancer cells. Macrophages can quickly and directionally migrate to pathological sites where specific chemokines are secreted, which allows them to serve as targeted drug delivery vehicles [70,71]. There are two types of macrophages that target M1 and M2 tumor cells, called Tumor Associated Macrophages (TAMs). TAMs are mainly induced by IL-6 and IL-10. They protect cancer stem cells, paving the way for metastasis, and weaken protective adaptive immunity [72]. During tumor initiation, TAMs exhibit properties characteristic of M1 macrophages, which condition the activation of pro-inflammatory intracellular pathways dependent on transcription factors (NF-KB). In turn, with the development of cancer, these cells transform into M2 macrophages, that inhibit signaling pathways determining expression of pro-inflammatory cytokines. TAMs also inhibit T cell activity thereby promoting tumor progression [70,72,73,74]. In studies of skin cancer and lung metastases in experimental mice, it was found that triterpenoid compounds (e.g., oleanolic acid) reduced the transcription factor STAT3 (Signal Transducers and Activators of Transcription) and inhibit macrophage polarization towards M2. These compounds also increased sensitivity of cancer cells to the action of anti-cancer drugs: Adriamycin and cisplatin [73].

## 6. Unfavorable Effects of Carbon Nanoparticles

In order to safely use nanoparticles in therapy, it is necessary to determine their potential toxicity and their elimination routes more precisely. Naota et al. [75] showed that the absorption of fullerenes from the alimentary and respiratory tract is low. They conducted studies on mice by administering fullerene (0.7 nm) intratracheally at the dose of 25 mg/kg and 40 mg/kg. Under the light microscope, they observed aggregates of fullerene molecules in capillaries and lymph nodes immediately after application. Using the electron microscope, an increased number of pinocytotic vesicles and caveolae were also observed in type I pneumocytes. This means that due to diffusion and pinocytosis, fullerenes can cross the air-blood barrier [75]. These particles can move to other tissues with blood. The highest concentration of fullerenes was observed in the liver, and smaller amounts in the spleen, kidneys and lungs. There are also indications of adverse effects of nanotubes on biological systems. It has been observed that they can change the structure of some proteins, which is associated with their malfunction. This leads to formation of inflammatory factors and the body’s immune response, and cell dysfunction [76]. Morimoto et al. [77] showed that exposure of rats to MWCNTs (mean fiber size: diameter 63 nm, length 1.1 μm) at the concentration of 0.37 mg/m^3^ for 4 weeks caused development of inflammatory changes in lungs. Fibers of multi-wall carbon nanotubes were dispersed compared to single-walled ones, which formed larger aggregates. That could have influenced the toxic effect [77]. In another experiment, MWCNTs (diameter 158 nm, length 5.8 μm) were administered intratracheally to rats at concentrations of 0.5 mg/m^3^, 2.5 mg/m^3^ and 25 mg/m^3^. Animals were followed for 90 days after the end of the exposure. Small clusters of fibers outside the lung were observed, depending on concentration. In rats exposed to 2 higher concentrations (2.5 mg/m^3^, 25 mg/m^3^), inflammation occurred in the bronchioloalveolar region and clusters of fiber-filled alveolar macrophages, while no toxic effects were observed in animals exposed to the lowest concentration [19,78]. Further studies on toxicity of carbon nanotubes were carried out by Kim et al. [79]. They investigated biotin-modified and anticancer drug conjugated SWCNTs and MWCNTs coupled to Fe nanoparticles loaded with doxorubicin. Normal human peripheral blood lymphocytes were treated with three different concentrations of MWCNTs (12.5; 25; 50 mg/mL) and incubated with cells for 48 h. A significant delay in growth of lymphocytes and damage to their DNA were observed. Increased levels of ROS have also been noted [66]. Chernova et al. [80] proved that CNTs can affect the body similarly to asbestos fibers, and therefore may pose an inhalation hazard similar to asbestos. Studies confirmed that the instillation of long nanotube fibers into the pleural cavity of mice induced mesothelioma, that has pro-oncogenic effect during the latency period of disease progression. Long-term activation of pro-oncogenic signaling pathways, increased proliferation and DNA oxidative damage, create a common molecular signature for the pathology caused by long nanotubes. Visalli et al. [81] found that MWCNTs induced DNA damage in neuronal cells and led to the formation of reactive oxygen species triggering a pro-inflammatory response. Inflammation of neurons was confirmed by the presence of elevated levels of pro-inflammatory cytokines TNFα, IL-1β, IL-6 and cytokines [81]. Fresta et al. [82] investigated the effect of ND at a low concentration (2 µg/mL) on viability of BU-2 microglial cells and A549 vesicular epithelium, and on production of NO and ROS. They observed a 5% decrease in the count of cells. In both cell lines, non-cytotoxic concentrations of ND increased NO and ROS production. Therefore, they caused formation of oxidative/nitrosative stress. Karpeta-Kaczmarek et al. [83] also observed the effect of two concentrations of ND: 20 and 200 mg/L of food administered to the Acheta Domesticus cricket. After 7 weeks, at the applied concentration of 200 mg/L, an increase in the parameters of oxidative stress was observed, i.e., an increase in the activity of catalase and glutathione peroxidase, and an increase in the level of heat shock proteins. After 14 weeks, the analysis of single and double-stranded DNA damage using the comet test also showed DNA damage in insects that received the higher dose of ND. These results may suggest that toxicity of ND is concentration dependent.

Opposite results were reported by Moche et al. [27] who conducted the research on HBE14 bronchial epithelial cell lines and T84 colon cancer cells. NDs of various diameters of 20, 50 and 100 nm was added to the medium and cells were incubated for 4 h. DNA damage was assessed using a comet test and a micronucleus test. The obtained results did not confirm any significant genotoxic effect on any of the tested cell lines. Toxicological data are presented in Table 1. The results of biological response studies to nanoparticles in in vivo and in vitro conditions confirm that both the toxic effects of these nanoparticles on biological systems and their absence depend on size and concentration of nanoparticles.

## 7. Conclusions

Currently, carbon nanoparticles play a large role as carriers of various types of drugs, and also have applications in other fields of medicine, e.g., in tissue engineering as they are used to reconstruct bone tissue. They also contribute to the early detection of cancer cells, thus, they can act as markers in imaging diagnostics. Their antibacterial and anti-inflammatory properties are also known. This feature is particularly important in dental implantology, where various types of bacterial infections and implant rejection often occur. The search for newer and more effective treatments may lead to future use of nanoparticles on a large scale. Therefore, there is a need for further, more in-depth research and analyses of their potential applications, but also of their toxicity, so that, apart from their numerous positive applications, the risk of exposure of normal cells to these particles was as low as possible. Based on the literature review, it can be seen that despite many reports indicating the advantages of using carbon nanoparticles in targeted therapy, diagnostics, and teranostics, the observed toxic effects indicate caution and careful consideration of the selection of the type of nanoparticle, its dimensions, concentration, method of administration or attachment of functional groups or the use of complexation with molecules increasing the biocompatibility of carbon nanoparticles.

## Figures and Tables

**Table 1:** Toxicity of carbon nanoparticles on biological systems depending on the dose and size in vitro and in vivo; D—diameter, L—length of nanoparticles.

Biological System	Administration Route	Type of Nanoparticle	Dose (concentration) and Size of Nanoparticles	Mechanism of Toxicity	Reference
Mice	Alimentary, respiratory	Fullerene C_60_	25 mg/kg 40 mg/kg	Accumulation in capillaries, lymph nodes, liver, spleen, and lungs	[75]
Rats	Intratracheal Inhalation	MWCNT	12 nm (D), 0.4 µm (L) 67 nm (D), 4.0 µm (L) 0.5 mg/m^3^ 1.5 mg/m^3^	Increased levels of DNA damage, inflammation. Fibrosis only at the higher dose of 67nm/4.0 µm NTs	[84]
Rats	Intratracheal Inhalation	MWCNT	63 nm (D), 1.1 μm (L); 0.2 mg/rat 1.0 mg/rat 0.37 mg/m^3^	Inflammatory changes in lungs	[77]
Rats	Intratracheal	MWCNT	158 nm (D), 5.8 μm (L) 0.5 mg/m^3^ 2.5 mg/m^3^ 25 mg/m^3^	Accumulation of fibers in lungs, at the two highest concentrations, inflammation of the respiratory tract	[78]
Mice	Intratracheal	CNT	Long-fiber carbon nanotubes; 5 µg/mouse	Induction of pleural mesothelioma, an asbestos-like hazard	[80]
Neuronal cells	Incubation	MWCNT	15-30 nm (D), 10-20 µm (L) or 0.2-1 µm (L); 15-20 layers; 12.5 µg/mL, 25 µg/mL	DNA damage, ROS formation, neuronal inflammation, elevated levels of cytokines TNFα, IL-1, IL-3, IL-6.	[81]
Human peripheral blood lymphocytes	Incubation	SWCNT/Biotin MWCNT/Fe/DOX	12.5mg/mL 25mg/mL 50mg/mL	Delayed lymphocyte growth and damage to their DNA, increased levels of ROS	[79]
Microglial cells BU-2 Alveolar epithelial cells A549	Incubation	ND	2 µg/mL	5% decrease in cell viability Increased production of NO and ROS	[82]
Bronchial epithelium cell lines HBE14; Colon carcinoma cells T84	Incubation	ND	20nm, 50nm, 100nm (D); 12.5–100 µg/mL	No genotoxic effects were observed in any of the lines	[27]
House cricket Acheta Domesticus	Alimentary	ND	20mg/L in food 200mg/L in food	No toxic effects observed Increased parameters of oxidative stress, catalase, glutathione peroxidase and heat shock proteins, DNA instability	[83]

## Data Availability

Not applicable.
